# Technology of Processing Plant Extracts Using an Aluminometasilicate Porous Carrier into a Solid Dosage Form

**DOI:** 10.3390/pharmaceutics14020248

**Published:** 2022-01-21

**Authors:** Klára Kostelanská, Slavomír Kurhajec, Sylvie Pavloková, David Vetchý, Jan Gajdziok, Aleš Franc

**Affiliations:** 1 Department of Pharmaceutical Technology, Faculty of Pharmacy, Masaryk University, Palackého třída 1946/1, 612 00 Brno, Czech Republic; 507255@muni.cz (K.K.); pavlokovas@pharm.muni.cz (S.P.); vetchyd@pharm.muni.cz (D.V.); franca@pharm.muni.cz (A.F.); 2 Department of Pharmaceutical Technology, Pharmacognosy and Botany, University of Veterinary Medicine and Pharmacy, Komenského 73, 041 81 Košice, Slovakia; slavomir.kurhajec@uvlf.sk

**Keywords:** porous carriers, liquisolid systems, magnesium aluminometasilicates, adsorption, plant extracts, antioxidant activity

## Abstract

A method of preparing tablets called liquisolid technique is currently emerging. In these formulations, an important role is played by porous carriers, which are the basic building blocks of liquisolid systems (LSSs). The most common are microcrystalline cellulose (MCC), magnesium aluminometasilicates, silica aerogels, mesoporous silicates, clays, etc. In this study, magnesium aluminometasilicate is used to prepare modified LSS formulations with plant extracts as model drugs dissolved in water (W) or ethanol (E). The modification involves drying tablets in a microwave (MW) and hot air dryer (HA) for a specified period. Powder blends and tablets were evaluated for physical properties, and their antioxidant activity (AA) was measured in a modified dissolution by ferric reducing antioxidant power assay (FRAP). PLS and ANOVA were used to compare tablets properties depending on the composition and technology. The experiment is based on a previous one, in which the plant extracts were processed into tablets using a similar method. Therefore, extending the study to include more plants and the robust statistical evaluation and comparison of the products was a procedure to justify the suitability of the presented method for a wide range of liquid plant extracts. As a result, we obtained tablets with excellent physical properties, including a short disintegration and dissolution, which is problematic in tableted extracts.

## 1. Introduction

The growing interest in natural medicines is reflected in the research and development of dosage forms. Liquid natural extracts are usually hydrophilic and therefore chemically and microbially unstable. Therefore, their processing into solid dosage forms is promising. However, the processing of larger amounts of dried extracts is limited due to their poor flowing properties and compressibility. An original technology based on the liquisolid system (LSS) using a porous carrier and hydrophilic solvents, subsequently evaporated from the compacts, proved promising for plant extracts with antioxidant activity (AA). The work demonstrated the technological possibilities of preparation on a limited number of model extracts. A more comprehensive study of whether the technology itself affects AA and whether AA was retained concerning different plant species was necessary due to significant differences in the composition of each extract (Table 1) [1]. The first work was focused mainly on plant materials; this work focuses on the theoretical justification of the porous material used.

Porous carriers are essential structural materials used to prepare innovative drug formulations. Their promising properties include a homogenous structure, high specific surface area (SSA), appropriate pore size necessary for drug incorporation, and advantageous technological properties, such as flow, compression, etc. [11,12,13]. The porous structure of these materials allows the adsorption of liquid drugs and their subsequent release in a predictable manner. Due to the ability to adsorb the drug, these substances have found application in liquisolid systems preparation, liquid systems in the solid phase, or the preparation of solid self-emulsifying systems [11]. After incorporating the liquid component into the porous structure of the carrier, a solid system with properties appropriate for further technological processing is formed. The liquid penetration through the porous material depends on the volumetric and molecular properties of the liquid and the geometrical and surface properties of the carrier. When a porous-material-containing dosage form comes into contact with a solvent (e.g., GI fluid), the active ingredient is washed or dissolved from the surface. Subsequently, the drug diffuses through the pores filled with the dissolution medium [11].

A few promising materials have been tested as carriers in pharmaceutical research in recent years. However, most of them were discarded mainly due to their non-biocompatibility or limited final processability [14].

Microcrystalline cellulose (MCC) is one of the most used carriers in pharmaceutical technology due to its high stability, proven applicability, and good availability. MCC was used as a carrier in several studies. For example, the study of Sanka et al. aimed to increase the solubility of clonazepam. Poorly water-soluble clonazepam was formulated into a LSS using propylene glycol as the solvent, Avicel^®^ PH 102 as the carrier, and Aerosil^®^ 200 as the coating material. This study showed a decrease in the crystallinity of clonazepam in a LSS, which was reflected in an improvement in its solubility [15]. MCC (Avicel^®^ PH 102) as carrier was used by Chella et al. in a study to enhance the dissolution profile of valsartan. After 30 min of the dissolution study, twice more valsartan was released than from commercial tablets [16]. Other cellulose derivatives, such as hypromellose (HPMC) and methylcellulose (MC), were also investigated as carriers in solid self-emulsifying systems (S-SMEDDS) [17,18,19].

Another porous carrier is colloidal silicon dioxide [20]. In a study by Bhagwat et al., Aerosil^®^ 200 was involved as a carrier in the formulation of S-SMEDDS with the poorly water-soluble drug telmisartan. Increased dissolution rate and higher bioavailability of the drug were proven [21].

Several studies used other materials as carriers, e.g., dibasic calcium phosphate anhydrous (DCPA), calcium silicate, silica aerogel, mesoporous silicates, or clays. Fujicalin^®^ (DCPA) was used as a carrier in the study by Krupa et al. to improve the solubility of furosemide by LSS formulations. The results showed a positive effect of the carrier on the dissolution rate of the drug [22]. Florite^®^ RE (FLR, calcium silicate) was used as a porous carrier in the study by Sharma et al., dealing with the formulation of accelerated release tablets of meloxicam. The results showed that FLR was characterized by a large SSA available for drug adsorption (~130 m^2^·g^−1^). After incorporating the drug into its porous structure, meloxicam crystallized, increasing its solubility [23].

Silica aerogel was used as a carrier for the poorly soluble drugs ketoprofen and griseofulvin. The drugs were first dissolved in supercritical carbon dioxide. Subsequently, they were absorbed onto the carrier, and formulation solubility was compared to pure crystalline drugs. In both cases, the release was more advantageous than in the crystalline form [24].

In the study by Javadzadeh et al., mesoporous silicates were successfully used as sorbents of a liquid phase excess after their incorporation into an MCC carrier in a LSS formulation containing carbamazepine. The results showed an increased solubility and dissolution rate of the LSS tablets [25].

Ibuprofen was intercalated into the structure of montmorillonite. Dissolution tests showed that clay material could be used as a carrier for the sustained release of the drug [26]. Kevadiya et al. incorporated lidocaine into the structure of montmorillonite by intercalation. The formulation provided the pH-dependent controlled release of lidocaine [27].

However, the most used porous carriers are magnesium aluminometasilicates. These materials are available in alkaline or neutral and powdered or agglomerated forms. Most often, Neusilin^®^ is used with SSA = 110–300 m^2^/g and adsorption capacity for liquids 1.0–3.1 m^2^/g. There are four types of Neusilin^®^ (threegranular, one powder). Neusilin^®^ is an inert amorphous material prepared by spray drying (agglomerated types) [28].

Neusilin^®^ is an important excipient for various technological processes related to solid dosage forms. It can improve the flow properties of powders, act as a binder, improve tablet hardness or play an important role as an adsorbent in solid dispersions or as a carrier in S-SMEDDS and LSSs. It also helps to protect the drug from moisture, prevents caking, and can stabilize the drug from transitioning from a crystalline to an amorphous form [29]. In the study of Zeman et al., Neusilin^®^ US2 was used in combination with MCC to increase the porosity of pellets usable as carriers in tubes to detect nerve agents. The presence of Neusilin^®^ led to a statistically significant increase in the strength, intraparticle porosity, and sphericity of the prepared pellets [30]. The work of Hentzschel et al. aimed to substitute the carrier Avicel^®^ by an excipient with higher SSA and suitable flow properties. The porous carriers Avicel^®^ PH 102, Fujicalin^®^ (dicalcium phosphate), and Neusilin^®^ US2 (agglomerated magnesium aluminosilicate) were tested with tocopherol acetate as the model drug. This research proved that the mentioned carriers have different adsorption capacities. Using Neusilin^®^ as a carrier and coating material, the tocopherol acetate adsorption capacity was increased by 47% [31]. In the study by Jadhav et al., Neusilin^®^ US2 was described as a suitable carrier for progesterone tablets. Furthermore, the proper flow and compressibility properties were obtained [32]. Kamel et al. prepared SEDDS with rutin. As excipients, the study used emulsifier, co-emulsifier, and oil. The prepared emulsion system was adsorbed by three carriers: Neusilin^®^ US2, Fujicalin^®^, and F-melt^®^. A powder blend composed of Neusilin^®^ US2 reported the best flow properties (2:1—carrier:liquid phase). During the dissolution, 90% of the drug was released in the first 15 min [33].

From the above comparison of the review [34] and experimental study [2], magnesium aluminometasilicate Neusilin^®^ appeared to be a suitable porous tablet carrier for the preparation of LSSs. Although LSSs are mainly used to increase the solubility and dissolution of lipophilic substances, tablets with a high content of hydrophilic substances, such as plant extracts, can also be prepared. This claim is supported by a study by Kurhajec et al., where Neusilin^®^ was successfully used with plant extracts by modified LSS technology [1]. The difference with conventional LSSs was that an aqueous or ethanolic solution with a concentrated plant extract was used instead of a drug dissolved in a water-miscible liquid. A porous magnesium aluminometasilicate (Neusilin^®^ US2) was used as a carrier to incorporate concentrated aqueous and alcoholic extracts from *Aronia melanocarpa* Michx. Elliott, *Crataegus leavigata* Poir. DC, and *Rosa canina* L. (Rosaceae). The wet mixtures were coated and mixed with colloidal silicon dioxide, croscarmellose sodium, and magnesium stearate and tableted. The evaporation of used solvents was the final preparation step (different from LSSs). The tablets were dried by two different techniques: microwave drying (MW) and hot air drying (HA) at two different times. The ethanol used in the preparation procedure increased the hardness, prolonged the disintegration, and slightly increased the water absorption of tablets. MW led to tablets with higher friability and longer disintegration. All tablets were characterized by rapid disintegration and dissolution, contributing to optimal conditions for immediate drug release and the extracts’ better bioavailability [1].

This work deals with the similar preparation technique of tablets containing fruit extracts of *Prunus padus* L., *Prunus spinosa* L., and *Rubus fruticosus* L. (Rosaceae) (Table 1). The mentioned plants are rich sources of anthocyanins, flavonoids, organic acids, vitamins, and other substances that give them an antioxidant, anti-carcinogenic, anti-inflammatory effect, and many other benefits [2,3,4,5,6,7,8,9,10]. Extracts from the plants could also supplement diabetic patients, such as other natural drugs the authors deal with.

The research expands the existing work by the mutual comparison and robust statistical evaluation (PLS and ANOVA) of the physico-chemical properties of the powder blends and tablets. It evaluates the antioxidant activity of all used drugs with FRAP (ferric reduction antioxidant power assay). For the mutual comparison, the evaluations are performed by the same methods using the same equipment and conditions as in the previous work [1].

## 2. Materials and Methods

### 2.1. Materials

Liquid extracts from dried fruits of *Prunus padus* L., *Prunus spinosa* L., and *Rubus fruticosus* L. (F-Dental, Hodonín, Czech Republic) were used. Ethanol 96% (Mikrochem, Pezinok, Slovakia) and purified water prepared by reverse osmosis (Ph. Eur.) were used as solvents. As a porous carrier, Neusilin^®^ US2 (Fuji Chemical Industry Co, Ltd., Toyama, Japan) was used. Aerosil^®^ 200 (Evonik Industries AG, Essen, Germany) is the coating material. The other excipients used for tablets formulation were: filler Avicel^®^ PH 101 (FMC Bio-Polymer, Ireland), lubricant magnesium stearate (Zentiva, Prague, Czech Republic), and disintegrant Vivasol^®^ (JRS Pharma, Rosenberg, Germany). A 2% ethanolic solution of brilliant green (Dr. Kulich Pharma, Hradec Králové, Czech Republic) was used to determine water absorption and wetting time. AA was determined using ferric chloride hexahydrate (Mikrochem, Pezinok, Slovakia), ferrous sulfate heptahydrate (Mikrochem, Slovakia), hydrochloric acid (Centralchem, Bratislava, Slovakia) and 2,4,6-tris(2-pyridyl)-s-triazine (Sigma-Aldrich, St. Louis, MI, USA). All reagents for AA determination were of analytical quality.

### 2.2. Methods

#### 2.2.1. Preparation of Extracts

Vacuum concentrated plant extracts were prepared by long-term maceration (7 days) with ethanol (50% *w*/*w*) as an extracting agent by the same procedure described in the study of Kurhajec et al. [1].

#### 2.2.2. Preparation of Powders and Formulation of Tablets

The whole process of the preparation of powder blends and LSS tablets with plant extracts (materials, solvents, equipment, temperature, length of drying, etc.) was the same as described in the study of Kurhajec et al. [1]. The composition of powder blends was mentioned in Table 2. The samples of powder blends were marked following their composition (name of the extracted plant and the first letter of used solvent, respectively). Samples of tablets were marked based on their drying method (name of the extracted plant, the first letter of used solvent, the type of drying method, and length of drying, respectively).

#### 2.2.3. Evaluation of the Powder Blends

According to Ph. Eur., the flow rate through the orifice of powder blend (flowability) was performed with a flowability tester (Ing. Havelka, Brno, Czech Republic) [35]. The whole procedure was described in the study of Kurhajec et al. [1]. The measurements were performed three times, and the results were presented as mean values ± standard deviations.

A fixed glass funnel and free-standing cone were used to determine the angle of repose, subsequently calculated according to Ph. Eur. [1,35]. The measurements were performed three times, and the results were presented as mean values ± standard deviations.

The pycnometric density of the powder blends was determined using the gas displacement technique with a helium pycnometer (Pycnomatic Act, Porotec, Haan, Germany), according to Ph. Eur. [35,36].

Values measured from bulk, tapped, and pycnometric densities were used to determine the Hausner ratio (HR), compressibility index (CI), intra- and inter-particle porosity. The resulting values were calculated according to Ph. Eur. [35].

The angle of slide is a specific parameter used especially for measuring the flow properties of powder blends formulated by the LSS technique. The whole procedure was described in the study of Kurhajec et al. [1]. The measurements were performed three times, and the results were presented as mean values ± standard deviations.

The loss of drying of powder blends was evaluated by a halogen moisture analyzer (Mettler Toledo, HX204, Im Langacher Greifensee, Switzerland) under the given conditions. The whole procedure was described in the study of Kurhajec et al. [1]. The measurements were performed three times, and the results were presented as mean values ± standard deviations.

#### 2.2.4. Physico-Mechanical Evaluation of the Tablets

According to Ph. Eur., the physico-mechanical properties of the tablets (hardness, weight uniformity, and height) were tested by an automatic tablet testing system (WHT-1, PharmaTest, Hainburg, Germany) [35].

According to Ph. Eur., ten randomly selected tablets were used for the friability test, placed into the plastic drum of the friability tester (Tar 10, Erweka, Langen (Hessen), Germany) [35].

A disintegration test was performed with six randomly selected tablets from each batch and a disintegration test apparatus (ZT4, Erweka, Langen (Hessen), Germany) [35].

The wetting time and water absorption ratio (WA) of LSS tablets were evaluated according to the studies of Vraníková et al. and Kurhajec et al. [1,37].

For liquisolid tablets, tests, such as pycnometric density and loss of drying, were performed on the same principle as for powder blends. The whole procedure was described in the study of Kurhajec et al. [1]. The measurements were performed three times, and the results were presented as mean values ± standard deviations.

The porosity of oblong tablets was calculated from the pycnometric density of tablets (*ρ_p_*) and their powder blends (*ρ_pm_*) according to Equation (1) [1,30]:

(1) $$P=\left(1-\frac{\rho_{p}}{\rho_{pm}}\right)\cdot 100$$

#### 2.2.5. Scanning Electron Microscope (SEM)

The morphology of liquisolid tablets in fracture was determined by SEM (MIRA, Tescan, Brno, Czech Republic. The whole procedure was described in the study of Kurhajec et al. [1]. The obtained signals of the samples were produced by secondary electrons (SE) at 3 kV voltage and 500× magnifications.

#### 2.2.6. Dissolution and Evaluation of Antioxidant Activity

The dissolution was carried out using a paddle apparatus (SR8 Plus, Hanson, Neperville, USA) under the given conditions: purified water 500 mL, 37 ± 0.5 °C, paddle 50 rpm, sampling time 5, 10, 15, 20 and 30 min. Each sample was immediately analyzed for AA by the FRAP method [37]. The whole procedure was described in the study of Kurhajec et al. [1].

#### 2.2.7. Data Analysis

Analysis of variance (ANOVA) statistical testing combined with the partial least squares (PLS) in software R, version 4.1.0, were employed [38]. The results of the PLS2 algorithm for multivariate data were visualized via correlation loadings plot, which enables to observe relationships between the process and formulation variables (solvent, drying setup) and the selected tablet characteristics. The ANOVA was applied to confirm or disprove the statistical significance of the studied effects (corresponding *p*-value is given in parentheses for each discussed tablet property throughout the Results and Discussion Section). A detailed methodology is available in the previous article [1].

AA maintenance is an essential parameter for assessing tablet quality. Therefore, a comprehensive comparison of the percentual decrease in AA after drying depending on the process/formulation parameters, including tablets prepared within the previous article [1], was performed by ANOVA.

## 3. Results and Discussion

This study aims to find or affirm the appropriate composition of liquisolid blends and tablets suitable for plant extract incorporation. The choice of porous carrier and suitable excipient was important. Tablets were formulated using an innovative technological method based on the drying of LSS using various formulation/method parameters: extract type (*Prunus padus* L.—PrunusP, *Prunus spinosa* L.—PrunusS, and *Rubus fruticosus* L.—Rubus), used solvent (ethanol—E, water—W), drying method (hot air dryer—HA, microwave oven—MW), and drying time (60 or 180 min during HA, 1 or 3 min during MW) were evaluated for physico-mechanical properties and also for AA (antioxidant activity).

Water (W) and ethanol (E) were chosen as solvents due to easy evaporation without the residues. W and E were not presented in the final tablets and therefore did not have a cosolvent role as in the conventional LSS method.

### 3.1. Evaluation of the Powder Blends

The flowability of prepared tablet powder blends ranged from W (15.92 ± 0.66 s/100 g Blank_W) to E (46.01 ± 0.35 s/100 g PrunusP_E). The results show that W powder blends had better flowability (Table 3). The lubricating properties of W were caused by its reduction in electrostatic forces and particle friction [39]. In the case of W (15.92 ± 0.66 s/100 g) and E (25.75 ± 0.31 s/100 g), powder blends without an extract had better flowability than blends with extract—W (19.97 ± 2.04 s/100 g Rubus_W) and E (46.01 ± 0.35 s/100 g PrunusP_E). According to these data, the plant extract’s presence negatively affected the flowability behavior of the powder blends (Table 3), which was also observed in the study of Kurhajec et al. [1].

The determination of the angle of repose (Table 3) did not show any effect of used plant extract or type of solvent. The measured values of angle of repose (31.52 ± 1.65° PrunusS_W–39.28 ± 0.78° Blank_W) corresponded to excellent (25–30°), good (31–35°) or fair (36–40°) flow properties, as described in Ph. Eur. [35], and were comparable to another study of powder blends with plant extracts (30.54 ± 2.52–39.28 ± 0.78°) [1]. The placebo blends contained more humidity than the blends with extracts. The loss of drying confirmed this statement. It could be explained by an increased amount of W adsorbed on the surface of the particles, which increased the capillary bridges’ strength, resulting in a negative effect on the flow [30].

In the case of pycnometric density (Table 3,) blends with an extract showed higher values (1.5567 ± 0.0016 g/cm^3^ Rubus_E—1.6313 ± 0.0042 g/cm^3^ PrunusP_E) than samples without an extract (1.4764 ± 0.0006 g/cm^3^ Blank_E—1.4974 ± 0.0007 g/cm^3^ Blank_W). The presence of a plant extract affected the pycnometric density of the powder blends. The study of Gumaste et al. confirmed that increased moisture could cause a decrease in pycnometric density. The reason is filling pores with liquid [40].

Values of intra-particle (69.25% Blank_E—78.63% PrunusP_E) and inter-particle (77.72% Blank_E—82.87% PrunusP_E) (Table 3) porosity did not show any differences related to the used extract or type of solvent. The measured data corresponded to the previous works [41,42].

The determination of the compressibility index (CI) (16.33% PrunusS_E—27.54% Blank_E) and Hausner ratio (HR) (1.20 PrunusS_E/Rubus_E—1.38 Blank_E) (Table 3) showed that powder blends reported good, fair, or passable flow character, according to Ph. Eur. [35]. Samples Blank_E (CI 27.54%, HR 1.38), PrunusP_W (CI 22.95%, HR 1.23) and Rubus_W (CI 26.05%, HR 1.26) showed poor flow character. Finally, all the measured values of powder blends were comparable with other studies (CI 11–30%, HR 1.1–1.4), although they used chemically uniform drugs and different solvents [37,43]. The powder blends with the extract *Rubus fruticosus* showed cohesive forces (more pronounced in this plant extract [44]), increasing the HP and CI.

The determination of the angle of slide (Table 3) showed that the sample PrunusP_W (33.08 ± 0.53°) had an optimal angle of slide recommended by Spireas (33°). Other samples, such as PrunusS_E and Rubus_E (both 34.30 ± 1.53°), also came close to the recommended value [45]. The other blends had (30.70 ± 0.58° PrunusS_W—44.00 ± 3.00° Blank_W) slightly lower and higher values, indicating worse flow properties. A similar effect was observed in the angle of repose, where the strength of capillary bridges negatively affected flow properties [30].

The values measured for loss of drying (Table 3) showed that W blends (13.85 ± 1.75% Rubus_W—26.28 ± 0.19% Blank_W) had higher moisture content than E blends (11.40 ± 0.26% PrunusP_E—19.23 ± 0.09% Rubus_E). This finding was also observed in the study of Jagia et al. [46].

### 3.2. Physico-Mechanical Evaluation of the Tablets

Table 4 (for W) and Table 5 (for E) summarized data related to the physico-mechanical parameters of the tablets formed by the modified LSS technique.

**Figure 1:**
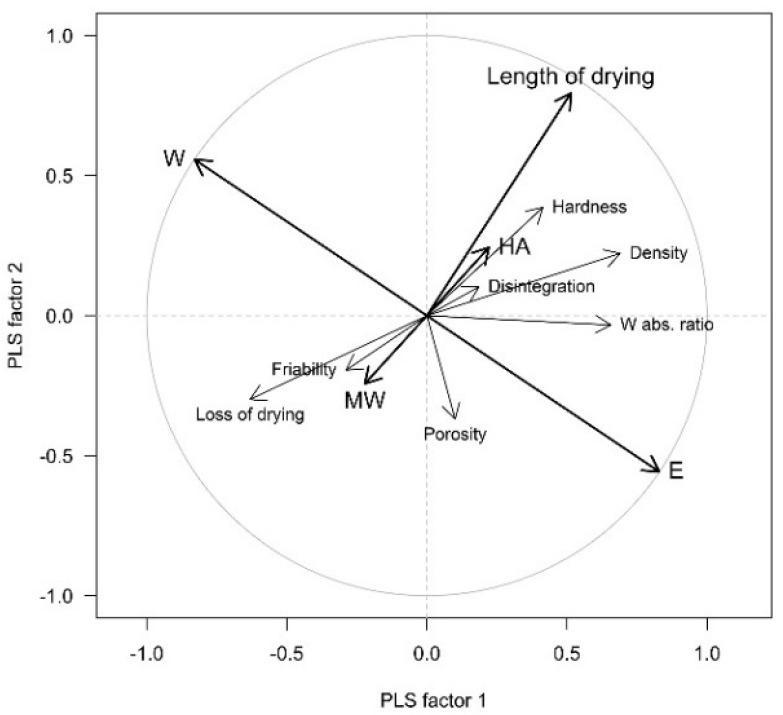
Correlation loadings plot (PLS); arrows for input variables (preparation parameters) depicted by bold lines; arrows for output variables (physico-mechanical parameters) represented by thin lines.

In the PLS correlation loadings plot (Figure 1), the outputs of this testing and tablet process/formulation parameters (drying setup and used solvent) were expressed in mutual relations. The following tablet properties were excluded from the analysis: height and weight (comparable values for W and E extracts), wetting time (due to missing values for some samples, especially for Rubus_E samples), and weight uniformity (statistically insignificant effects were proven by ANOVA). The remaining variables listed in Table 4 and Table 5 were visualized and discussed using PLS. Plant extract presence and type (Blank, PrunusP, PrunusS, and Rubus) were not examined in more detail because they did not significantly clarify and simplify the data structure in the correlation loadings plot. Moreover, only dried tablets were included in the PLS analysis to interpret results better.

The first and second PLS components comprised 62.1% and 29.7% of the total variability in the X matrix and in the Y matrix, respectively, which can be considered sufficient for screening purposes. Two main correlation trends in data are visible (Figure 1). The distinction between ethanolic and aqueous solutions was dominant in the direction from the upper left quadrant to the lower right quadrant. In the perpendicular direction, i.e., in the direction from the upper right quadrant to the lower left, the effect of drying length in combination with the minor impact of drying type (MW and HA) could be seen.

Thus, this PLS depiction could show a masking effect for values that increased with the length of drying and, at the same time, were higher for MW dried samples than for samples dried by HA (or in the opposite case) because corresponding arrows were directed against each other. The more dominant effect of these two influenced prevails in the PLS plot. The mentioned masking issue was specifically related to disintegration time and friability. However, the results were sufficiently commented on one-dimensional data visualization (not included in the article) and supported by ANOVA statistical testing.

The type of used solvent had an impact on tablets quality. The water absorption ratio (*p* < 0.001) of E tablets (7.42 ± 5.40% Blank_E_Undried—40.43 ± 28.29% Blank_E_MW_3 min) was higher than in W tablets (14.53 ± 10.46% PrunusP_W_HA_180 min—52.61 ± 18.29% Blank_W_HA_60 min). In the study of Pabari and Ramtoola, tablets containing W as a solvent had a lower water absorption ratio [47]. Due to their highly porous structure and zero W content, they could absorb more liquid. The previous statement confirmed that E tablets had also a higher pycnometric density (*p* < 0.001) (1.4764 ± 0.0006 g/cm^3^ Blank_E_Undried—1.7958 ± 0.0076 g/cm3 Rubus_E_MW_3 min) and porosity (*p* < 0.001) (3.17% Blank_E_MW_3 min—17.71% Blank_E_Undried) than the W samples. The pycnometric density increased with decreased moisture in the tablets. According to the measured data, it was evident that the W tablets had a higher moisture loss of drying (*p* < 0.001) than E samples (apparent from Figure 1 and Table 4 and Table 5). In the case of another physical property of the tablets, it could be stated that E tablets had a slightly higher hardness (*p* = 0.002) than the W tablets. In the study of Tank et al., the same phenomenon was observed. Tablets containing hydro-alcoholic solvent had greater hardness than tablets formulated using W-based solvents. The residual liquid phase could affect hydrogen bonds between cellulose particles, which leads to a higher hardness of dosage form [48,49]. The effect of used solvent (E vs. W) on the disintegration time (*p* = 0.073), friability (*p* = 0.764) and wetting time (*p* = 0.249) was assessed as statistically insignificant.

Regarding the type of drying method used, it was observed that MW tablets had higher friability (*p* = 0.006) and longer disintegration time (*p* < 0.001) than HA tablets. The direction and length of the respective vectors in the PLS correlation loadings plot (Figure 1) were also affected by the more pronounced influence of the drying length of these quantities. Most samples showed lower friability values than is the limit for uncoated tablets (max 1%) as defined in Ph. Eur. In the case of dried tablets with an extract, pharmacopoeial limits were confirmed for all samples [35]. HA tablets had a higher water absorption ratio than MW samples. This applies particlely to W samples (*p* = 0.040). For E samples, it was without a significant effect (*p* = 0.423). The type of drying had no statistically significant effect on hardness (*p* = 0.128), density (*p* = 0.220), porosity (*p* = 0.518), loss of drying (*p* = 0.180) and wetting time (*p* = 0.308).

With the increase in length of drying, hardness (*p* < 0.001), disintegration time (*p* < 0.001) and pycnometric density (*p* < 0.001) increased, and all these vectors were closely correlated (Figure 1). These results correlate to the study of Chowhan et al., where it was mentioned that increasing the length of drying tablets increases hardness and disintegration time [50]. All measured samples showed much lower values than the limit for uncoated tablets (max 900 s) as defined in Ph. Eur. [35]. A more significant increase in hardness with drying time was observed for W tablets than for E tablets. From these findings, it could be deduced that the amount of W in the tablets had a decisive effect on the hardness. The explanation may be that the hardness of the formulations decreased with the increase in moisture to a certain point, from which the increasing moisture increased the hardness [51,52]. The water absorption ratio (*p* < 0.001) and friability (*p* = 0.021) were also positively correlated with drying length (Figure 1). However, the vector of friability points to the other side due to opposing effects—a higher friability in MW samples than in HA samples. This was also the reason for the lower vector length for disintegration time. It could be explained by the content of magnesium stearate, which exhibited polymorphism and contributed to the formation of new solid bridges during high temperatures [53]. The wetting time for HA samples (*p* = 0.015) and loss of drying (*p* < 0.001) decreased with higher drying length, as is evident from the negative correlation of corresponding arrows (Figure 1). The wetting time for MW samples (*p* = 0.309) and porosity (*p* = 0.347) were not statistically influenced by the length of drying.

Finally, the influence of plant extract presence was visible on the values of friability (*p* = 0.032) and loss of drying (*p* < 0.001), which were higher for blank than for plant extract samples. The values of hardness (*p* < 0.001), porosity (*p* = 0.022) and density (*p* = 0.020) were lower for blank than for plant extract samples. Thus, it could be stated that the presence of the plant extract had a positive effect on the friability of the tablets. The correctness of this assumption was also indicated by the available knowledge about the ability of plant extracts to act as a binder, which increased the hardness and reduced the friability of tablets [54]. The type of plant extract has no significant effect on friability (*p* = 0.182) and loss of drying (*p* = 0.672). The dependencies of other quantities varied greatly.

### 3.3. Scanning Electron Microscopy (SEM)

**Figure 2:**
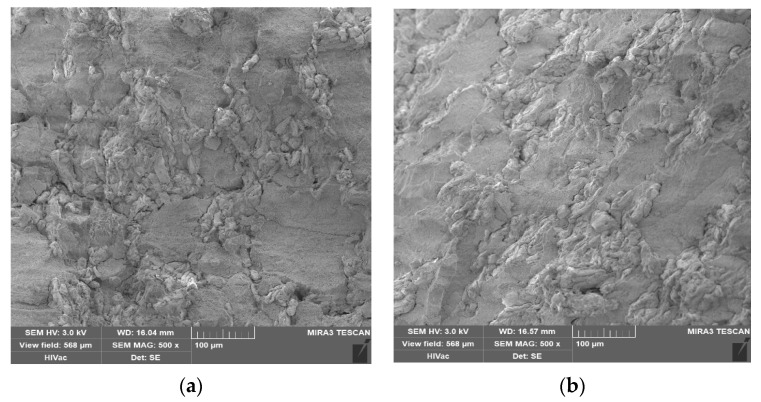
Images of Blank_E_HA_180 min (**a**) and Blank_E_MW_3 min (**b**).

**Figure 3:**
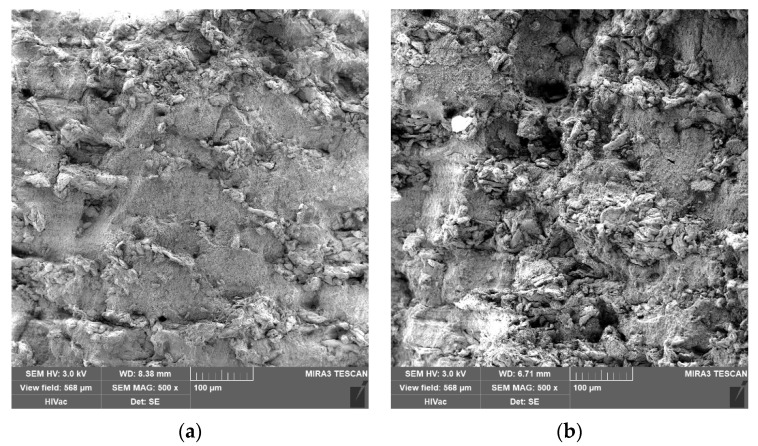
Images of Blank_W_HA_180 min (**a**) and Blank_W_MW_3 min (**b**).

**Figure 4:**
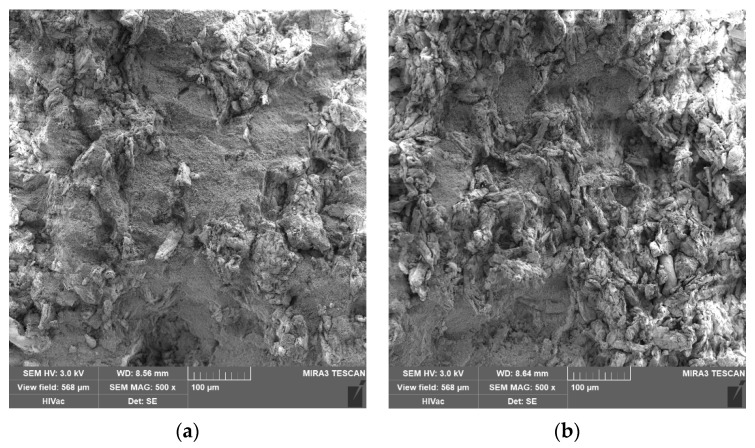
Images of PrunusP_E_HA_180 min (**a**) and PrunusP_E_MW_3 min (**b**).

**Figure 5:**
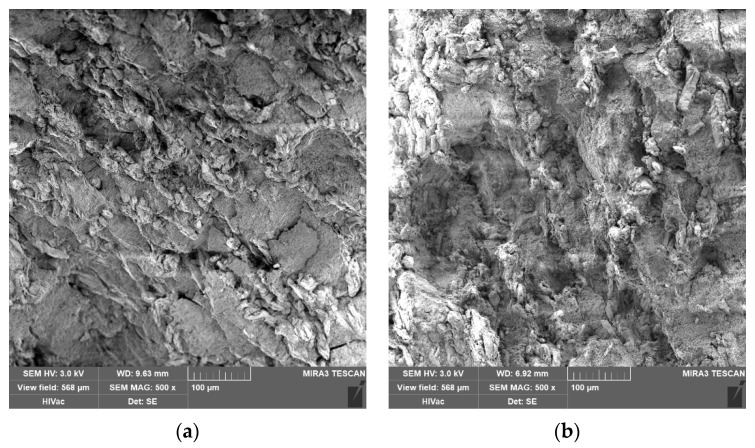
Images of PrunusP_W_HA_180 min (**a**) and PrunusP_W_MW_3 min (**b**).

The SEM images (Figure 2, Figure 3, Figure 4 and Figure 5) showed little differences between E and W tablets. W tablets showed some structural collapses (cracks) emerging during the HA drying. The evaporation and deformation of tablets could be caused by long drying. The observed changes varied on the composition, size, and shape of the dried material, the drying length, and the drying method [52,55]. More differences revealed images without plant extracts (2) and with plant extracts (3–5). It was visible that the presence of an extract in the tablet reduced conductivity, which was confirmed by the differences between the individual images.

### 3.4. Dissolution and Antioxidant Activity of the Prepared Liquisolid Tablets

The blank tablets (without extract) showed low AA (Table 6) due to the presence of reducing monosaccharides arising from the partial hydro-thermal degradation of polysaccharide excipients (Avicel^®^ PH 101) [1]. The AA value was slightly higher in W tablets because water represented a more suitable environment for hydrolysis than E [56,57].

A slightly increased AA was observed in the dried placebo tablets. The AA value increased with HA drying time for E tablets, while it decreased with MW drying time (*p* < 0.001). This trend was the opposite for W tablets (*p* < 0.001). The observed differences may be caused by the unequal values of the vaporization heat of the used solvents and the different energy efficiency of the used drying methods. The measured AA values of the placebo tablets were subtracted from the AA values of the relevant tablet batches containing the plant extracts.

The largest differences in AA were between the values of the blank tablets and the tablets containing plant extracts. Flavonoids, anthocyanins and other plant substances, mainly from polyphenols, are responsible for the high AA of tablets containing extracts (Table 7) [58,59,60]. The AA of tablets decreased in the following order: PrunusS > Rubus/PrunusP > blank (*p* < 0.001). For W tablets, AA also decreased in the order PrunusS > Rubus > PrunusP > blank, but for tablets with E, it decreased in the order PrunusP > PrunusS > Rrubus > blank (*p* < 0.001 in both cases).

The AA values of tablets with PrunusS (W 0.804 ± 0.004–1.201 ± 0.004; E 0.506 ± 0.002–0.665 ± 0.009) and Rubus extract (W 0.668 ± 0.003–0.853 ± 0.010; E 0.444 ± 0.007–0.642 ± 0.009) were higher when W was used as the solvent, whereas for the PrunusP extract (W 0.501 ± 0.001–0.727 ± 0.004; E 0.584 ± 0.012–0.650 ± 0.001), this observation was the opposite (*p* < 0.001 for all mentioned trends). The observed differences were probably caused by the different content of antioxidants in individual plant extracts as well as the different solubilization and stability of individual substances in aqueous or ethanolic environments (sensitivity to oxidation, hydrolysis, bacterial contamination subsequently followed by bacterial enzymes activity, etc.) [61,62]. MW drying appeared a more advantageous drying process to achieve higher AA for PrunusS (MW 0.630 ± 0.004–1.103 ± 0.004; HA 0.506 ± 0.002–1.045 ± 0.001) and PrunusP (MW 0.518 ± 0.004–0.643 ± 0.007; HA 0.501 ± 0.001–0.601 ± 0.011) tablets (*p* < 0.001 for both cases). The drying method did not affect the tablets with Rubus (*p* = 0.361).

Hihat et al. and Lanasno et al. pointed out the safety of MW drying of antioxidant drugs based on polyphenols. Hayat et al. explained the possible increase in the AA of plant material loaded by MW radiation by releasing and activating bounded phenolic compounds [63,64,65].

With drying time, the AA decreased for tablets containing PrunusS extract (1 min 0.647 ± 0.004–1.103 ± 0.004/3 min 0.630 ± 0.004–0.832 ± 0.002; 60 min 0.572 ± 0.002–1.045 ± 0.001/180 min 0.506 ± 0.002–0.804 ± 0.004) and *p* extract (1 min 0.622 ± 0.002–0.643 ± 0.007/3 min 0.518 ± 0.004–0.633 ± 0.010; 60 min 0.559 ± 0.003–0.601 ± 0.011/180 min 0.501 ± 0.001–0.584 ± 0.012) (*p* < 0.001 in both cases), but the length of drying did not have a significant effect on the Rubus tablets (*p* = 0.335). Kurhajec et al. and Volf et al. pointed to a reduced number of substances with AA in plant extracts after long-term heating [1,66].

The higher AA of the tablets that contained an aqueous Rubus extract was achieved when dried in the HA (HA 0.795 ± 0.008–0.804 ± 0.005; MW 0.668 ± 0.003–0.680 ± 0.004). A slight increase in AA with drying time in the HA was observed in the tablets containing E extracts of Rubus (*p* < 0.001 in both cases).

**Scheme 1:**
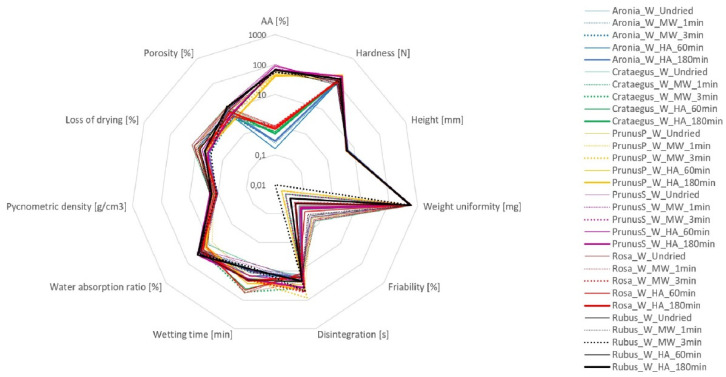
Graph of the physico-mechanical properties and AA of the tablets with W plant extracts.

**Scheme 2:**
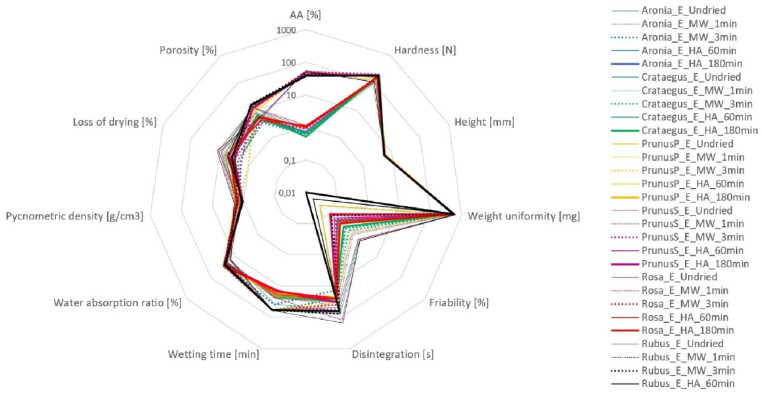
Graph of the physico-mechanical properties and AA of the prepared tablets with ethanolic plant extracts.

**Scheme 3:**
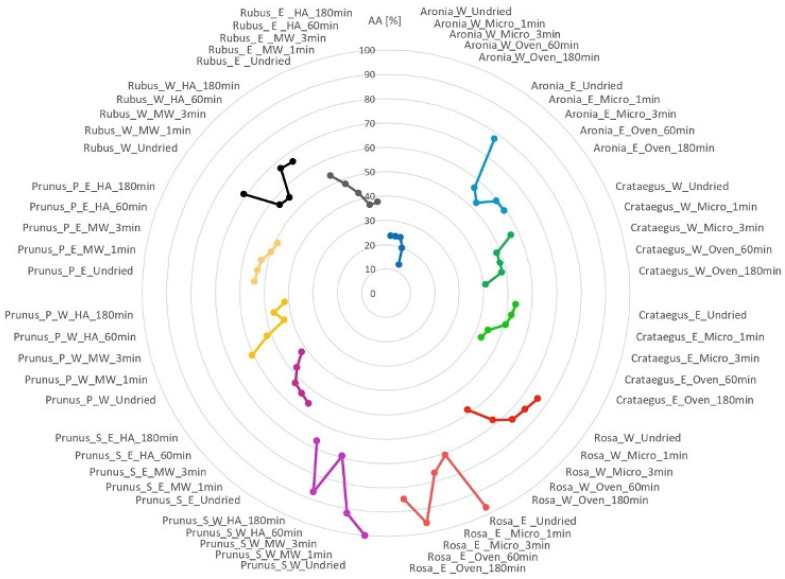
Graph of the tablets’ AA.

The comparison of the physico-mechanical properties and AA of the prepared modified liquisolid tablets and tablets from another study with plant extracts, prepared by Kurhajec et al. [1], is presented ], is presented in Scheme 1, Scheme 2 and Scheme 3. The differences in AA were caused by the inter-individual variability of individual fruit types [67]. When comparing the AA values of tablets after and before drying, a decrease in units up to tens of percent was found. The plant extract type found a statistically significant effect on the AA percentage decrease (*p* = 0.045 when analyzing the whole dataset and *p* < 0.05 for all tested data subgroups: W and E samples, HA and MW samples, and combinations of these conditions). In terms of drying setup, MW is a more suitable method of tablet drying to maintain their AA value. It was found that MW samples generally showed a lower decrease in AA (by 14% of AA on average) than the HA samples (by 18.8% on average) (*p* = 0.003). When all extract types are included in the analysis, an insignificant effect of used solvent (*p* = 0.344) and length of tablet drying (*p* = 0.219) on AA decrease was confirmed.

## 4. Conclusions

The modified LSS method using a metasilicate carrier (Neusilin US2) with the final drying of tablets is suitable for incorporating liquid plant extracts into the solid dosage form. All formulated tablets showed an appropriate disintegration time and fast drug release expressed by a sufficient AA over time.

This work summarizes the AA and physical characteristics of tablets prepared from six plants processed into twelve plant extracts (W and E) and offers a comprehensive statistical evaluation of their AA concerning the variables of the preparation methods. Comparing previously published research and the current work enables us to draw valid conclusions.

The obtained data showed that E tablets had a higher water absorption ratio, pycnometric density, porosity, slightly higher hardness, and prolonged disintegration time. W tablets showed a higher loss of drying. MW drying produced tablets with a higher friability and disintegration time, while HA tablets had a higher water absorption ratio. The length of drying increased hardness, disintegration time, and pycnometric density. The drying time positively influenced water absorption ratio and friability.

The type of used plant material was confirmed as the main factor influencing the AA of the tablets. Despite the interindividual variability between different plant extract types, it was found that MW drying generally leads to the better maintenance of AA after drying than HA drying. However, the effect of the used solvent and length of drying on the percentage decrease in AA of the dried tablets compared to non-dried tablets was evaluated as insignificant.

## Figures and Tables

**Table 1:** Overview of the used medicinal plants, main active substances, and their therapeutic use.

Medicinal Plant	Active Substances	Therapeutic Use	Literature
*Prunus padus* L.	anthocyanins, cyanogenic glycosides, flavonoids (kaempferol and quercetin derivatives); phenolic acids (chlorogenic, neochlorogenic, and caffeic acid), coumarins (aesculetin, umbelliferone, scopoletin)	antioxidant, antibacterial, anti-inflammatory, anti-nociceptive, therapy of swelling	[2,3,4]
*Prunus spinosa* L.	anthocyanins (cyanidin and peonidin derivatives), flavonoids (quercetin, kaempferol), tannins, organic acids, pectin, saccharides, vitamin C, and cyanogenic glycosides (amygdalin, prunasin) contained in seeds	antioxidant, antibacterial, astringent, antidiarrheal, anti-inflammatory, digestive disorders, diuretic, cardiovascular diseases	[5,6,7]
*Rubus fruticosus* L.	anthocyanins (cyanidin derivatives), flavonoids (rutin, quercetin), organic acids (ferulic acid, caffeic acid, p-coumaric acid, ellagic acid), catechin, epicatechin, saccharides, vitamins	antioxidant, anti-carcinogenic, anti-inflammatory, prevention, and supportive therapy of diabetes	[8,9,10]

**Table 2:** Composition of the powder blends.

	LSS— ETHANOLIC Extract		LSS— AQUEOUS Extract	
	[g]	[%]	[g]	[%]
Plant extract	50.75	12.50	50.75	12.50
Ethanol 96%	50.75	12.50	0.00	0.00
Purified water	0.00	0.00	50.75	12.50
Neusilin^®^ US2	101.50	25.00	101.50	25.00
Aerosil^®^ 200	2.04	0.50	2.04	0.50
Avicel^®^ PH101	188.78	46.50	188.78	46.50
Magnesium stearate	4.06	1.00	4.06	1.00
Vivasol^®^	8.12	2.00	8.12	2.00
Total	406.00	100.00	406.00	100.00

**Table 3:** Physical evaluation of the powder blends.

Sample of the Tablet Blends	Flowability	Angle of Repose	Angle of Slide	Loss of Drying	CI	HR	Intra-Particle Porosity	Inter-Particle Porosity	Pycnometric Density
	(s/100 g)	(°)	(°)	(%/1 g)	(%)		(%)	(%)	(g/cm3)
Blank_E	25.75 ± 0.31	36.26 ± 2.48	39.70 ± 0.58	16.17 ± 2.18	27.54	1.38	69.25	77.72	1.4764 ± 0.0006
Blank_W	15.92 ± 0.66	39.28 ± 0.78	44.00 ± 3.00	26.28 ± 0.19	17.14	1.21	74.09	78.53	1.4974 ± 0.0007
PrunusP_E	46.01 ± 0.35	32.16 ± 0.54	31.70 ± 1.53	11.40 ± 0.26	19.85	1.25	78.63	82.87	1.6313 ± 0.0042
PrunusP_W	16.19 ± 3.72	33.08 ± 0.53	33.70 ± 0.58	15.07 ± 0.70	22.95	1.23	75.42	80.01	1.6088 ± 0.0008
PrunusS_E	20.32 ± 1.90	33.07 ± 1.41	34.30 ± 1.53	13.68 ± 0.77	16.33	1.20	77.34	81.04	1.5691 ± 0.0065
PrunusS_W	17.56 ± 2.92	31.52 ± 1.65	30.70 ± 0.58	15.31 ± 1.02	20.97	1.21	75.91	80.08	1.5897 ± 0.0018
Rubus_E	31.55 ± 1.55	37.96 ± 0.47	34.30 ± 1.53	19.23 ± 0.09	16.48	1.20	78.00	81.63	1.5567 ± 0.0016
Rubus_W	19.97 ± 2.04	36.86 ± 0.83	31.00 ± 1.00	13.85 ± 1.75	26.05	1.26	75.18	80.31	1.5699 ± 0.0025

**Table 4:** Physico-mechanical evaluation of tablets with W plant extracts.

Sample	Hardness	Height	Weight Uniformity	Friability	Disintegration	Wetting Time	Water Absorption Ratio	Pycnometric Density	Loss of Drying	Porosity
	(N)	(mm)	(mg)	(%)	(s)	(min)	(%)	(g/cm^3^)	(%)	(%)
Blank_W_Undried	110.7 ± 5.5	5.41 ± 0.03	655.1 ± 1.5	1.85	26.0 ± 2.0	3.20 ± 0.15	20.80 ± 17.19	1.4902 ± 0.0009	22.29 ± 1.08	16.94
Blank_W_MW_1 min	127.5 ± 11.0	5.45 ± 0.05	622.4 ± 3.3	2.22	36.0 ± 2.0	8.30 ± 1.36	21.36 ± 14.62	1.5183 ± 0.0002	20.26 ± 1.63	15.37
Blank_W_MW_3 min	159.2 ± 7.5	5.31 ± 0.02	534.9 ± 2.1	0.67	26.0 ± 2.0	53.45 ± 8.05	16.19 ± 12.01	1.6710 ± 0.0038	7.34 ± 0.96	6.86
Blank_W_HA_60 min	151.6 ± 12.2	5.36 ± 0.03	588.9 ± 4.9	0.03	28.0 ± 1.0	9.22 ± 0.43	52.61 ± 18.29	1.5734 ± 0.0019	14.54 ± 1.15	4.27
Blank_W_HA_180 min	145.1 ± 11.9	5.29 ± 0.02	500.1 ± 1.2	0.36	39.0 ± 2.0	15.36 ± 1.05	17.93 ± 12.94	1.7175 ± 0.0043	6.17 ± 0.23	12.30
PrunusP_W_Undried	107.7 ± 11.4	5.51 ± 0.12	639.1 ± 1.1	0.49	25.0 ± 1.0	13.45 ± 3.18	20.54 ± 12.48	1.5783 ± 0.0016	11.58 ± 2.08	12.55
PrunusP_W_MW_1 min	125.9 ± 3.1	5.41 ± 0.06	623.3 ± 4.7	0.50	45.0 ± 1.0	15.15 ± 0.40	7.97 ± 6.24	1.5938 ± 0.0011	10.24 ± 1.55	11.44
PrunusP_W_MW_3 min	211.3 ± 5.6	5.32 ± 0.05	566.1 ± 3.3	0.02	85.0 ± 4.0	19.55 ± 5.40	16.00 ± 10.84	1.6841 ± 0.0004	3.92 ± 0.01	5.07
PrunusP_W_HA_60 min	136.7 ± 13.7	5.46 ± 0.13	595.0 ± 5.9	0.02	29.0 ± 1.0	26.20 ± 9.23	16.46 ± 12.69	1.6369 ± 0.0021	7.47 ± 0.65	8.64
PrunusP_W_HA_180 min	214.3 ± 5.2	5.27 ± 0.05	567.9 ± 2.0	0.14	46.0 ± 1.0	20.30 ± 1.48	14.53 ± 10.46	1.6857 ± 0.0009	4.08 ± 0.17	3.92
PrunusS_W_Undried	98.4 ± 1.5	5.67 ± 0.03	654.0 ± 4.0	0.45	19.0 ± 1.0	5.43 ± 0.18	21.48 ± 14.63	1.5975 ± 0.0007	12.35 ± 2.04	12.03
PrunusS_W_MW_1 min	107.4 ± 2.8	5.71 ± 0.06	621.1 ± 5.1	0.33	25.0 ± 3.0	6.55 ± 0.44	24.86 ± 6.42	1.6356 ± 0.0003	9.60 ± 1.10	11.12
PrunusS_W_MW_3 min	188.5 ± 5.4	5.63 ± 0.06	565.5 ± 2.8	0.03	53.0 ± 2.0	9.12 ± 1.39	30.44 ± 7.70	1.7106 ± 0.0023	3.56 ± 0.15	6.13
PrunusS_W_HA_60 min	132.5 ± 5.7	5.60 ± 0.03	597.2 ± 3.3	0.17	22.0 ± 1.0	14.50 ± 7.58	28.89 ± 9.20	1.6637 ± 0.0006	6.13 ± 1.07	8.77
PrunusS_W_HA_180 min	192.3 ± 7.0	5.55 ± 0.03	566.2 ± 3.9	0.15	37.0 ± 1.0	19.10 ± 1.06	36.57 ± 6.62	1.7092 ± 0.0026	3.71 ± 0.19	6.05
Rubus_W_Undried	90.2 ± 0.9	5.57 ± 0.01	643.7 ± 3.4	0.38	20.0 ± 1.0	10.0 ± 1.48	26.02 ± 10.32	1.5888 ± 0.0017	12.09 ± 3.06	5.75
Rubus_W_MW_1 min	106.9 ± 5.0	5.53 ± 0.02	603.4 ± 1.3	0.33	17.0 ± 1.0	11.12 ± 1.23	24.27 ± 6.57	1.6379 ± 0.0018	8.87 ± 1.05	8.58
Rubus_W_MW_3 min	160.2 ± 4.5	5.45 ± 0.01	550.6 ± 6.0	0.01	49.0 ± 1.0	7.33 ± 1.25	33.74 ± 9.23	1.7112 ± 0.0028	2.84 ± 0.49	12.49
Rubus_W_HA_60 min	122.5 ± 5.5	5.47 ± 0.00	579.4 ± 3.5	0.09	23.0 ± 2.0	20.32 ± 2.06	30.26 ± 7.67	1.6672 ± 0.0018	7.21 ± 0.42	10.18
Rubus_W_HA_180 min	152.0 ± 6.4	5.42 ± 0.01	559.1 ± 5.5	0.05	22.0 ± 1.0	8.05 ± 0.48	36.07 ± 5.57	1.6968 ± 0.0018	4.95 ± 0.38	11.75

**Table 5:** Physico-mechanical evaluation of tablets with ethanolic plant extracts.

Sample	Hardness	Height	Weight Uniformity	Friability	Disintegration	Wetting Time	Water Absorption Ratio	Pycnometric Density	Loss of Drying	Porosity
	(N)	(mm)	(mg)	(%)	(s)	(min)	(%)	(g/cm^3^)	(%)	(%)
Blank_E_Undried	97.5 ± 6.5	5.63 ± 0.04	630.3 ± 1.4	2.11	49.0 ± 3.0	>60	7.42 ± 5.40	1.4764 ± 0.0006	15.58 ± 0.39	17.71
Blank_E_MW_1 min	119.1 ± 5.7	5.59 ± 0.04	570.8 ± 1.1	0.82	29.0 ± 7.0	34.00 ± 8.00	18.45 ± 13.51	1.6146 ± 0.0031	11.47 ± 0.17	10.01
Blank_E_MW_3 min	125.2 ± 4.4	5.51 ± 0.06	506.4 ± 1.7	0.43	35.0 ± 10.0	15.21 ± 1.28	40.43 ± 28.29	1.7372 ± 0.0044	4.62 ± 0.19	3.17
Blank_E_HA_60 min	117.8 ± 6.1	5.53 ± 0.06	537.9 ± 1.2	0.26	19.0 ± 1.0	18.19 ± 1.02	20.69 ± 13.87	1.6714 ± 0.0038	7.94 ± 0.24	4.68
Blank_E_HA_180 min	122.7 ± 4.6	5.51 ± 0.09	513.7 ± 1.2	0.36	23.0 ± 3.0	13.08 ± 0.43	29.82 ± 20.49	1.7102 ± 0.0023	5.16 ± 0.16	6.84
PrunusP_E_Undried	125.7 ± 8.4	6.23 ± 0.01	615.5 ± 5.5	0.70	34.0 ± 2.0	22.07 ± 2.04	25.73 ± 17.57	1.6397 ± 0.0042	6.90 ± 0.09	8.68
PrunusP_E_MW_1 min	133.9 ± 8.5	6.20 ± 0.03	613.5 ± 1.4	0.32	29.0 ± 2.0	19.38 ± 1.49	21.94 ± 15.39	1.6622 ± 0.0020	5.63 ± 0.12	9.37
PrunusP_E_MW_3 min	164.8 ± 19.4	6.10 ± 0.01	574.7 ± 2.1	0.23	34.0 ± 7.0	>60	26.74 ± 18.03	1.7942 ± 0.0040	0.99 ± 0.08	12.13
PrunusP_E_HA_60 min	157.3 ± 10.4	6.12 ± 0.02	609.4 ± 1.2	0.04	25.0 ± 4.0	20.35 ± 3.53	26.72 ± 18.63	1.6784 ± 0.0007	4.54 ± 0.14	10.78
PrunusP_E_HA_180 min	163,7 ± 13.0	6.12 ± 0.01	595.6 ± 1.3	0.26	23.0 ± 2.0	17.34 ± 2.41	29.38 ± 20.40	1.6838 ± 0.0040	3.21 ± 0.08	11.07
PrunusS_E_Undried	105.7 ± 1.9	5.70 ± 0.01	632.5 ± 1.1	1.92	124.0 ± 4.0	24.20 ± 0.35	16.44 ± 11.53	1.5691 ± 0.0065	10.66 ± 1.12	4.60
PrunusS_E_MW_1 min	175.7 ± 14.4	5.60 ± 0.11	581.0 ± 3.8	0.25	33.0 ± 9.0	17.20 ± 4.02	22.70 ± 15.64	1.6970 ± 0.0011	4.93 ± 0.17	11.76
PrunusS_E_MW_3 min	200.0 ± 16.5	5.71 ± 0.08	548.7 ± 4.4	0.18	55.0 ± 5.0	>60	26.92 ± 17.65	1.7354 ± 0.0016	1.81 ± 0.20	13.71
PrunusS_E_HA_60 min	172.8 ± 16.6	5.65 ± 0.13	579.3 ± 3.5	0.16	32.0 ± 5.0	14.10 ± 0.17	24.75 ± 16.98	1.6950 ± 0.0032	4.78 ± 0.10	11.66
PrunusS_E_HA_180 min	195.9 ± 19.3	5.50 ± 0.02	548.1 ± 4.8	0.11	33.0 ± 3.0	14.20 ± 0.35	27.52 ± 18.38	1.7106 ± 0.0040	3.21 ± 0.06	12.46
Rubus_E_Undried	111.0 ± 3.5	5.78 ± 0.02	636.4 ± 4.8	1.87	154.0 ± 30.0	>60	14.01 ± 9.79	1.5799 ± 0.0016	12.87 ± 1.13	5.22
Rubus_E_MW_1 min	163.0 ± 7.8	5.75 ± 0.04	570.7 ± 4.5	0.23	72.0 ± 2.0	>60	19.06 ± 15.01	1.7273 ± 0.0019	6.46 ± 0.25	13.31
Rubus_E_MW_3 min	178.1 ± 7.3	5.67 ± 0.02	534.9 ± 5.2	0.12	78.0 ± 3.0	>60	26.36 ± 18.66	1.7958 ± 0.0076	2.97 ± 0.13	16.61
Rubus_E_HA_60 min	179.9 ± 4.4	5.71 ± 0.03	559.0 ± 4.5	0.02	65.0 ± 2.0	>60	26.79 ± 18.66	1.7601 ± 0.0038	5.17 ± 0.18	14.93
Rubus_E_HA_180 min	189.5 ± 8.3	5.67 ± 0.01	548.1 ± 4.8	0.01	63.0 ± 6.0	>60	24.15 ± 15.83	1.7814 ± 0.0052	3.81 ± 0.09	15.94

**Table 6:** Average AA of blank tablets.

Sample	Average AA (FRAP) of Dissolution Media at Different Dissolution Times				
	5 min	10 min	15 min	20 min	30 min
Blank_W_Undried	0.000 ± 0.000	0.000 ± 0.000	0.000 ± 0.000	0.000 ± 0.000	0.000 ± 0.000
Blank_W_MW_1 min	0.095 ± 0.042	0.095 ± 0.042	0.095 ± 0.042	0.095 ± 0.042	0.095 ± 0.042
Blank_W_MW_3 min	0.143 ± 0.000	0.143 ± 0.000	0.143 ± 0.000	0.143 ± 0.000	0.143 ± 0.000
Blank_W_HA_60 min	0.167 ± 0.041	0.167 ± 0.041	0.167 ± 0.041	0.167 ± 0.041	0.167 ± 0.041
Blank_W_HA_180 min	0.095 ± 0.042	0.095 ± 0.042	0.095 ± 0.042	0.095 ± 0.042	0.095 ± 0.042
Blank_E_Undried	0.000 ± 0.000	0.000 ± 0.000	0.000 ± 0.000	0.000 ± 0.000	0.000 ± 0.000
Blank_E_MW_1 min	0.000 ± 0.000	0.000 ± 0.000	0.043 ± 0.014	0.068 ± 0.020	0.068 ± 0.020
Blank_E_MW_3 min	0.000 ± 0.000	0.000 ± 0.000	0.019 ± 0.017	0.019 ± 0.017	0.019 ± 0.017
Blank_E_HA_60 min	0.000 ± 0.000	0.000 ± 0.000	0.010 ± 0.017	0.010 ± 0.017	0.010 ± 0.017
Blank_E_HA_180 min	0.128 ± 0.017	0.128 ± 0.017	0.157 ± 0.017	0.176 ± 0.030	0.176 ± 0.030

**Table 7:** Average AA of tablets with plant extracts.

Sample	^1^ Average AA of Dissolution Media in Various Times of Sampling (FRAP)				
	5 min	10 min	15 min	20 min	30 min
Prunus_S_W_Undried	1.144 ± 0.004	1.178 ± 0.007	1.188 ± 0.004	1.202 ± 0.008	1.201 ± 0.004
Prunus_S_W_MW_1 min	0.813 ± 0.004	0.946 ± 0.004	1.095 ± 0.005	1.091 ± 0.008	1.103 ± 0.004
Prunus_S_W_MW_3 min	0.685 ± 0.020	0.717 ± 0.004	0.719 ± 0.005	0.781 ± 0.008	0.832 ± 0.002
Prunus_S_W_HA_60 min	0.971 ± 0.018	0.997 ± 0.011	1.012 ± 0.010	1.037 ± 0.007	1.045 ± 0.001
Prunus_S_W_HA_180 min	0.760 ± 0.016	0.781 ± 0.003	0.795 ± 0.014	0.800 ± 0.010	0.804 ± 0.004
Prunus_S_E_Undried	0.600 ± 0.010	0.662 ± 0.011	0.667 ± 0.008	0.662 ± 0.011	0.665 ± 0.009
Prunus_S_E_MW_1 min	0.557 ± 0.010	0.572 ± 0.002	0.590 ± 0.008	0.627 ± 0.013	0.647 ± 0.004
Prunus_S_E_MW_3 min	0.613 ± 0.005	0.660 ± 0.007	0.629 ± 0.003	0.629 ± 0.003	0.630 ± 0.004
Prunus_S_E_HA_60 min	0.465 ± 0.010	0.498 ± 0.005	0.514 ± 0.010	0.561 ± 0.005	0.572 ± 0.002
Prunus_S_E_HA_180 min	0.469 ± 0.008	0.478 ± 0.005	0.490 ± 0.009	0.500 ± 0.002	0.506 ± 0.002
Prunus_P_W_Undried	0.692 ± 0.009	0.696 ± 0.008	0.699 ± 0.011	0.725 ± 0.005	0.727 ± 0.004
Prunus_P_W_MW_1 min	0.536 ± 0.003	0.561 ± 0.003	0.580 ± 0.002	0.602 ± 0.003	0.622 ± 0.002
Prunus_P_W_MW_3 min	0.453 ± 0.003	0.454 ± 0.002	0.464 ± 0.004	0.516 ± 0.007	0.518 ± 0.004
Prunus_P_W_HA_60 min	0.433 ± 0.004	0.484 ± 0.006	0.511 ± 0.001	0.532 ± 0.003	0.559 ± 0.003
Prunus_P_W_HA_180 min	0.431 ± 0.002	0.441 ± 0.003	0.453 ± 0.004	0.490 ± 0.002	0.501 ± 0.001
Prunus_P_E_Undried	0.612 ±0.002	0.613 ± 0.002	0.639 ± 0.001	0.649 ± 0.001	0.650 ± 0.001
Prunus_P_E_MW_1 min	0.607 ±0.004	0.623 ± 0.003	0.636 ± 0.007	0.638 ± 0.007	0.643 ± 0.007
Prunus_P_E_MW_3 min	0.594 ± 0.003	0.591 ± 0.004	0.592 ± 0.006	0.627 ± 0.016	0.633 ± 0.010
Prunus_P_E_HA_60 min	0.524 ± 0.013	0.552 ± 0.008	0.590 ± 0.005	0.601 ± 0.008	0.601 ± 0.011
Prunus_P_E_HA_180 min	0.514 ± 0.007	0.545 ± 0.006	0.564 ± 0.014	0.576 ± 0.012	0.584 ± 0.012
Rubus_W_Undried	0.756 ± 0.004	0.777 ± 0.006	0.785 ± 0.008	0.852 ± 0.012	0.853 ± 0.010
Rubus_W_MW_1 min	0.545 ± 0.003	0.599 ± 0.009	0.619 ± 0.003	0.670 ± 0.005	0.680 ± 0.004
Rubus_W_MW_3 min	0.316 ± 0.034	0.452 ± 0.010	0.657 ± 0.012	0.666 ± 0.007	0.668 ± 0.003
Rubus_W_HA_60 min	0.692 ± 0.012	0.722 ± 0.004	0.758 ± 0.007	0.786 ± 0.008	0.804 ± 0.005
Rubus_W_HA_180 min	0.692 ± 0.006	0.756 ± 0.010	0.783 ± 0.006	0.790 ± 0.008	0.795 ± 0.008
Rubus_E_Undried	0.555 ± 0.007	0.564 ± 0.007	0.570 ± 0.007	0.636 ± 0.007	0.642 ± 0.009
Rubus_E_MW_1 min	0.426 ± 0.005	0.491 ± 0.009	0.526 ± 0.007	0.571 ± 0.009	0.574 ± 0.007
Rubus_E_MW_3 min	0.489 ± 0.003	0.504 ± 0.008	0.505 ± 0.006	0.509 ± 0.007	0.513 ± 0.008
Rubus_E_HA_60 min	0.343 ± 0.010	0.375 ± 0.006	0.403 ± 0.005	0.425 ± 0.006	0.444 ± 0.007
Rubus_E_HA_180 min	0.312 ± 0.009	0.406 ± 0.012	0.438 ± 0.007	0.452 ± 0.009	0.454 ± 0.007

^1^ AA is expressed in FRAP units. The values mentioned above AA were subtracted from the values of AA of the relevant placebo tablets.

## Data Availability

Data are contained within the article.
